# Lectins Are the Sparkle of Hope for Combating Coronaviruses and the Global COVID-19

**DOI:** 10.34172/apb.2022.030

**Published:** 2021-03-27

**Authors:** Heba Salah Abbas, Muddukrishnaiah Kotakonda

**Affiliations:** ^1^Microbiology Department, National Organization for Drug Control and Research (NODCAR), Egyptian Drug Authority, Giza, Egypt.; ^2^Department of Pharmaceutical Technology, Bharathidasan Institute of Technology, Anna University, Tiruchirappalli-620024.Tamilnadu, India.

**Keywords:** Coronaviruses, Genomic variations, Therapeutic options, Lectins

## Abstract

Today the crisis of coronavirus disease 2019 (COVID-19) pandemic represents a threatworldwide because it is a leading cause of human morbidity and mortality. Besides, it possessesa destroying impact on countries’ economies. Therefore, there is an urgent need for hardresearch work and global collaboration to find a potential therapy. In this review, structuralgenomic variations in COVID-19 and further therapeutic options of Coronaviridae family orCOVID-19 are expressed. Lectins are natural proteins, which can exist in algae, higher plants,banana, actinomycetes, fungi, and archaea, and they have antiviral properties. Griffithsin lectin,isolated from red algae, has noteworthy efficacy against lethal SARS-CoV infection, humancoronaviruses, and other animal coronaviruses. Furthermore, all mannose-specific plant lectinshave anti-coronaviruses properties except for garlic lectins. However, lectins from mushroomscan act as immunomodulators by activating T-lymphocyte or stimulating dendrites or cytokines.The lectin may hinder glucans on viral spike protein and prevent entry and the virus’s release.Lectin’s anti-coronavirus activities include a glimmer of hope to tackle the global COVID-19crisis and inspire more scientific work on carbohydrate-binding agents against SARS-CoV-2.

## Introduction

Viral respiratory tract infections worldwide pose a significant health threat and an immense economic burden for humans and animals. Human morbidity and death are caused worldwide by these respiratory infections, which cause serious public health problems, particularly for children, elderly persons, and people with immune problems.^1,2^ Indeed, the human airway can be contaminated by influenza viruses, coronaviruses, rhinovirus, adenoviruses, human metapneumovirus A1, and orthopneumoviruses such as the human respiratory syncytial virus. Children are the most susceptible group, as are elderly patients, and account for 95% and 40% of all virus-induced respiratory diseases.^3,4^ Besides, new respiratory viral agents are emerging periodically, triggering more severe effects of viral epidemics or pandemics, including neurological disorders.^5,6^ These peculiar events are usual when the species barrier is crossed in the animal storage system as an opportunity to adapt to newer environments and/or hosts by RNA viruses such as human coronaviruses.^7^ An unknown cause of pneumonia was first reported in Wuhan by the WHO Country Office in China in December 2019. In 2020, the outbreak was announced as an international public health emergency. The new coronavirus disease named COVID-19 was declared by the WHO. COVID-19 pandemic today represents a dramatic threat to life, and the virus spreads to all continents except Antarctica.^8^

Lectins are common natural proteins among higher plants, which are divided into structural and evolutionary proteins.^9^ Plant lectins have been reported to inhibit HIV replication in lymphocyte cell cultures over a decade ago by inhibiting virus-cell fusion.^10^

Lectins target a wide diversity of glycoproteins’ sugar moieties.^9^ The spike protein of SARS-CoV has 12 heavily glycosylated sites. Thus, the infectiveness of coronavirus can be predicted by lectins unique to glycans in the spike glycoprotein.^11^ Previous literature showed that algae lectin has noteworthy efﬁcacy against lethal SARS-CoV infections.^12^ This review article is discussing structural and genomic variations in COVID-19 (SARS-CoV-2). It also discusses further therapeutic options of the *Coronaviridae* family and lectins as potential therapeutic agents against human coronaviruses.

### Wuhan coronavirus or 2019-novel coronavirus (SARS-CoV2)

Wuhan, a growing business center in China, saw an outbreak of a novel coronavirus that killed over two millions people and infected more than hundred millions people till the date of this writing according to Worldometer (February 2021).^13^ This virus has been identified a member of the beta coronavirus group. The Chinese researchers have named the novel virus Wuhan novel coronavirus (2019-nCoV), or SARS-CoV-2 and the disease as COVID-19.^14^ The genetic recombination occurrence at S protein in receptor binding domain region of SARS-CoV-2 may improve the transmission ability of SARS-CoV-2 compared with SARS-CoV-1.^15^

### Source of COVID-19 infection

In China, Wuhan coronavirus pneumonia patients proposed visiting the seafood market, selling live animals or birds and animals for food. However, further studies have shown that some people contracted the infection without a visit to the seafood market, and the virus transmitted to more than 215 countries worldwide. Human to human spread of the virus takes place when the healthy person is in direct and close contact with the person who infected, exposed to cough, sneezing, air droplets, or aerosols. Through inhalation of the nose or mouth, aerosols can penetrate the lungs and human body.^16-18^

### Structure and genomic variation of COVID-19 (SARS-CoV-2)

COVID-19 has a typically spike protein coronavirus structure, as well as other polyproteins, nucleoproteins, and membrane proteins, such as RNA polymerase, 3-chymotrypsin-like protease, papain-like protease (PLpro), helicase, glycoprotein and accessory proteins.^19,20^

The 3-D structure in the receptor-binding domain region for the control of Van der Waals forces is present in the SARS-CoV-2 spike protein. The 394 glutamine residues in the receptor-binding domain region of the human angiotensin-converting enzyme 2 (ACE2) receptors are identified as essential lysine 31 residue.^21^ Resembling SARS-CoV, SARS-CoV-2 has also converting enzyme II (ACE2) as a cellular entry receptor by the angiotensin, proposing that the infection process of SARS-CoV-2 and SARS-CoV-2 into cells are identical.^22^ The recognized pathways of SARS-CoV-2 transmission in humans include inhalation small droplets carrying the virus, direct contact with virus carriers, being in contact with a virus contaminated surface, or aerosol transmission.^23^

Over 80% of the genome of SARS-CoV-2 has been informed to be the SARS-like bat CoV. The 5^՝^- open-ended reading frame1a/b is SARS-CoV-2’s main gene encoding the polyprotein 1ab, and 15 non-structural proteins. The main gene encodes for polyprotein1a, which also has 10 non-structural proteins.^24^ SARS-CoV-2 is located near the group of SARS-coronaviruses according to the evaluative tree.^25^ Recent literature on SARSCoV-2 has shown variation in the number of amino acids in 8b and 3c proteins, and lack of 8a protein.^24^ The SARS-CoV-2 spike glycoprotein consists of the SARS-CoV bat and an unknown beta CoV combination and a Single N501 T mutation in the spike protein of SARS-CoV-2 that may have greatly enhanced its ACE2 binding affinity.^21^ In addition, Wrapp et al showed that the spike protein of SARS-CoV-2 exhibits a higher binding affinity to the human ACE2 (ACE2-Peptidase) than does SARS-CoV. Also, the molecular dynamic stimulation was reported the strong binding of SARS-CoV2 to the human ACE2 due to the higher rapture force and larger puling work compared to SARS-CoV, which is determined by their electrostatic interaction.^22,26^ However, Walls et al reported that the variations between the two SARS- CoV2 and SARS- CoV are relatively small.^26^

## Therapeutic options for emerging viruses of coronaviridae family

Designing the suitable therapy depends upon the genomic structures of coronaviruses. The transcription and replication of CoV occur by RNA-dependent RNA polymerase (RdRp) and helicase. These enzymes are non-structural proteins which were produced as a result of cleavage of large replicase polyprotein 1a (pp1a) and pp1ab by two viral proteases; the PLpro and the 3C-like protease (3CLpro).^27^ Furthermore, the spike glycoprotein on the virus surface is responsible for the interaction between the virus and cell. The spike is composed of two subunits; amino-terminal receptor-binding S1 and carboxy-terminal membrane fusion S2. The entrance and fusion of viruses with the membrane require the breakage of protease location at the binding between S1 and S2. Monoclonal antibodies that target amino-terminal receptor-binding S1 and carboxy-terminal membrane fusion S2 will have antiviral activity against CoVs.^28^

Moreover, the pathogenicity and the host range of CoV depend on the host receptors. Thus, the therapeutic agents that target the host receptors are probable anti-CoV agent without causing any immunopathological influences for animal studies.^29^

There are two pathways for the host cell entry by CoVs:

1. Endosomal cell entry caused by low pH and the pH-dependent endosomal cysteine protease cathepsins.
2. Non-endosomal cell entry at plasma membrane caused by host proteases, such as transmembrane protease serine 2 that cleaves S into S1and S2 subunits and trigger S for virus entrance. The inhibition of these proteases can partially prevent the CoVs from the entrance of the host cells.^30,31^

**Figure 1:**
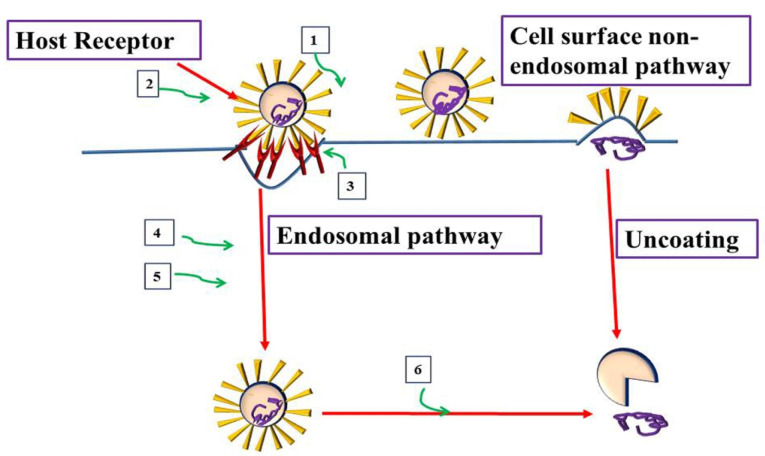
Virus and host-based treatments targeting attachment and entry of CoVs. Host-based treatment, and Virus based treatment.1. Monoclonal Antibodies targeting S1 or S2 viral subunits (REGN3051), 2. S2 Subunits Antiviral peptides (HR2P). 3. Monoclonal Antibodies, peptides against host receptor to inhibit entry (NAAE, YS110), 4. Clathrin inhibitors (Chlorpromazine, ouabain), 5. Cathepsins inhibitors (E64D), and 6. Endosomal acidification Inhibitors (chloroquine).

The possible virus and host-based treatments targeting the attachment and entry of CoVs were shown in Figure 1. When CoVs are within the host cell, the nucleocapsid and viral RNA in the cytoplasm begin to release. Upon wards, the 5ʹ- open-ended reading frame 1a/b is translated into p1a, and 1ab polyproteins. Cleavage of p1a and 1ab polyproteins generates several non-structural proteins including RNA dependent polymerase and helicase for replication and transcription processes. Standard CoV replication structures are formed following attachment of the hydrophobic domains of the CoV replication-transcription structure to the endoplasmic reticulum membrane.^32,33^

**Figure 2:**
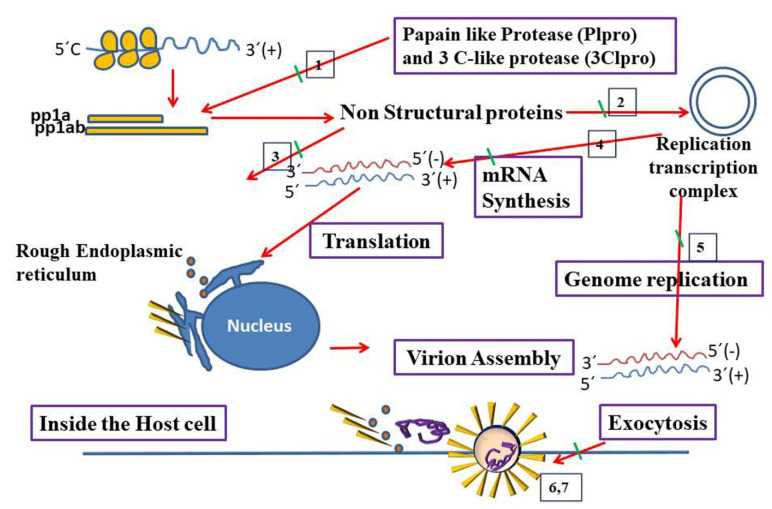
Virus and host-based treatments targeting transcription and replication of CoVs. Host- based treatment, and Virus based treatment. 1. Plpro inhibitors (Grlo617) and 3Clpro inhibitors (lopinavir), 2. Helicase inhibitors/RNA dependent/RNA polymerase Inhibitors (ribavirin), 3. CYP inhibitors (alisporivir), 4. SiRNAs against structural and accessory genes, 5. Membrane bound RNA inhibitors (K22)/Viral nucleic acid synthesis inhibitors, 6. Recombinant interferon Alfa, interferon beta, and 7. Interferon inducers (poly (1:C), nitazoxanide).

The transcription of the positive-strand RNA and synthesis of mRNA are essential for translation and generation of structural and accessory proteins. The nucleocapsid protein, genomic RNA, and structural proteins (helical nucleocapsid) form the assembled virionand the virion is then released outside the cell through exocytosis.^33,34^ Novel therapeutic agents that target membrane-bound RNA synthesis or structural genes, such as K22 and small interfering RNAs are potential treatments for CoVs as denoted in Figure 2.

## Lectins as potential therapeutic agents for combating coronavirus

Lectins are a diversity of non-immune glycoproteins with at least one non-catalytic domain that are reversibly bound to monosaccharide, oligosaccharide, and glycoconjugates. These proteins are also known as agglutinins and are commonly present in nature as monomers, homo-and hetero-dimers, and homo-and hetero-trimmer molecules. Lectins are common proteins, isolated from viruses, fungi, bacteria, invertebrates, single-cell organisms, livestock, and plants.^35^ Specific recognition according to a key lock model is promoted at lectin binding sites on the carbohydrates known as the carbohydrate recognition domain (CRD). In addition to the CRD, widely preserved in every type of lectins, weak chemical interactions are present.^36^ The diverse lectin activities are due to the binding carbohydrate as well as their molecular structure and specificity. Lectins are known to have an average size of proteins from 60 to 400 kDa.^37,38^

### Algae lectins as antivirals

For a long time, natural products have shown antitumor and antiviral activities. The proteinaceous leads contents of cyanobacteria *Nostoc ellipsosporum* and *Scytonemavarium* showed attractive antiviral activities. Cyanobacteria extracts possess antiviral proteins, such as cyanovirin-N (CV-N) and scytovirin of ~10 kDa, which may serve as anti-HIV.^39,40^

**Figure 3:**
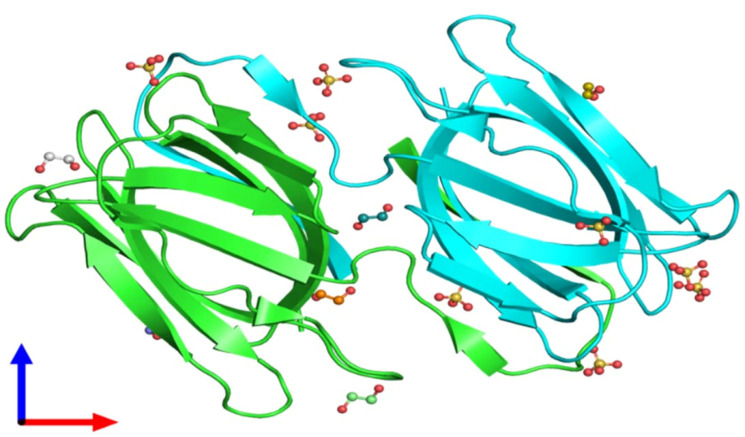
Crystal structure of unligand griffithsin using X-ray diffraction, 1.3 A° resolution, and assembly Composition (Source: Protein Data Bank in Europe).

Further literature showed a novel anti-HIV protein in the aqueous extract of red alga *Griffithsia*sp.that binds to various viral glycoproteins. This unique protein has a molecular weight of 12.7 kDa, with an unidentified amino acid at position 31(151 Da) (as illustrated by X-ray diffraction (Figure 3). Griffithsin is a diverse specificity lectin, which could be a potential antimicrobial to prevent virus infections.^41^

Previous reports showed that lectin griffithsin has noteworthy efﬁcacy against lethal SARS-CoV infection, human coronaviruses, and other animal coronaviruses. The potent antiviral activity against SARS-CoV was due to the carbohydrate speciﬁcity and the identified carbohydrate constituent of the SARS-CoV envelope glycoprotein S. Later, griffithsin treated infected animals were recovered, proposing that these cellular inﬁltrates may facilitate protective immunity to SARS-CoV. Additionally, the lower doses of griffithsin treatment could possess similar efficiency.^12^

### Plant lectins

Plant lectins are proteins which recognize and bind to carbohydrates reversibly. Müller et al documented that plant lectins formerly can inhibit virus replication through preventing virus-cell fusion.^42^ However, it was later shown that HIV particles’ fusion with their target cells was prevented by plant lectins.^10^

The in vitro inhibitor effects on HIV, cytomegalovirus influenza infection, respiratory syncytical virus infection, and influenza A-virus infection were also recorded by mannose/N acetyl-glucosamine agglutinin.^43^

A wide variety of plant lectins with different specificities were assessed for antiviral activity against SARS-Co-V using a colorimetric cell culture-based technique. Depending upon the essence of the sugar, the antiviral function of plant lectins differs considerably. The ability of plant lectins to interact with different targets is significant for inhibiting viral entry and depends on the position of the target glycans. Generally, all mannose-specific plant lectins have anti coronaviruses properties except for garlic lectins. The most antiviral lectin against SARS-CoV was the isolated mannose-specific plant lectin in leek, where the selectivity index was greater than 222, and 50% effective concentration was 0.45µg/ml. Furthermore, the N-acetyl glucosamine lectins content of stinging nettle and tobacco plant are also significantly active against the SARS-CoV and have selectivity index >77 and >59, respectively (Table 1).^44^

**Table 1:** Antiviral activity of plant lectins against SARS-CoV replication

**Lectin**	**Plant Species**	**EC**_50_ **(µg/mL)**	**CC**_50_ **(µg/mL)**	**SI**
**Mannose specific agglutinins**				
HHA	*Hippeastrum hybrid*	3.2 ± 2.8	> 100	> 31.1
GNA	*Galanthus nivalis*	6.2 ± 0.6	> 100	> 16.1
NPA	*Narcissus*	5.7 ± 4.4	> 100	> 17.5
APA	*pseudonarcissus*	0.45 ± 0.08	> 100	> 222.2
AUA	*Allium porrum*(Leek)	18 ± 4	> 100	> 5.5
CA	*Allium ursinum*	4.9 ± 0.8	> 100	> 20
LOA	*Cymbidium hybrid*	2.2 ± 1.3	> 100	> 45.5
EHA	*Listera ovata*	1.8 ± 0.3	> 100	> 55.5
Morniga M II	*Epipactis helleborine Morus Nigra*	1.6 ± 0.5	> 100	> 62.5
**N-acetyl glucosamine specific agglutinins**				
Nictaba	*Nicotiana tabacum*	1.7 ± 0.3	> 100	> 58.8
UDA	*Urtica dioica*	1.3 ± 0.1	> 100	> 76.9
Mannose/N-acetyl glucosamine specific agglutinins				
Cladistris	*Cladastris lutea*	7.4 ± 0.2	> 100	>13.5
**Galactose N-acetyl galactosamine (1,3) Galactose>Galactose N-acetyl galactosamine>Galactose-specific agglutinins**				
IRA	*Iris hybrid*	2.2 ± 0.9	50	22.7
IRA B	*Iris hybrid*	4.4 ± 3.1	36	8.2
IRA r	*Iris hybrid*	3.4 ± 2.0	55	16.2

EC_50_: 50% effective concentration, CC_50_: 50% cytotoxic concentration, SI: selectivity index.

In addition, the cytotoxicity and antiviral activity of plant lectins in Vero and CrFK cells were evaluated, and the toxicity of plant lectins in CrFK cells increased compared to VeroE6 cells.^44^

Only after the lectin was added during the infection, the intracellular viral RNA load decreased, thus showing the plant lectins interfere with the initial stage of the viral replication cycle. The entrance of SARS-CoV typically begins when the virus is attached to ACE2 cell receptors. The binding of the receptor then leads to the conformational modification of the protein of the viral envelope and causes the virus and cell membranes to fuse. The spike glycoprotein is necessary for coronavirus entrance. Therefore, the viral entry process is essential for the entry of antivirals or inhibitors.^44,45^

Earlier literature demonstrated the importance of spike protein not only for viral fusion but also for viral evolution. Since the replication cycle of SARS-CoV takes six hours to complete in Vero E6 cells and the plant lectins interact both at the virus’s entrance and release.^46^

### Banana lectin

Banana lectin is a Jacalin-related lectin family member. Musa acuminate, isolated from banana fruits, is also related to the structures of mannose. The natural lectin is a dimer made up of two identical 15 kDa units with 141 amino acids and two sites of sugar-binding.^47,48^

Banana’s lectin can inhibit diverse in-vitro HIV-1. Like the carbohydrate proteins, banana lectin prevents infection with HIV at virus entry by binding on the highly glycosylated gp120 to high-mannose structures and preventing the virus from sticking to them in a concentration-dependent manner. The new banana lectin has the potential to be used as a viricide drug.^49^

### Fungal lectins

Fungi are known for long periods as a rich source of diverse bioactive compounds. Inulin specific lectin (ABL) had been purified from the *Agaricusbitorquis* dried fruiting bodies. The ABL is a monomeric molecular protein of 27.6 kDa that is unlike other lectins of the *Agaricus* genus. The N-terminal amino acid sequence (EYTISIRVYQTNPKGFNRPV) is special and extremely related in the mushroom lectins. ABL inhibits the reverse transcriptase activity in HIV-1 and leukemia cell proliferation (L1210), with a 50% cytotoxic inhibition value of 4.69 and 4.97 μM, respectively.^50^

Furthermore, lectins from mushrooms can act as immunomodulators by activating T-lymphocyte or stimulating dendrites or cytokines, as in Table 2.^51^ Nowadays, the transport of Ca^2+^ by SARS-CoV has been documented to activate inﬂammasome stimulation. It has been proposed that the cytokine is related to infected patients by SARS-CoV-2.^52^

**Table 2:** Immunomodulatory of mushroom lectins

**Lectin**	**Source**	**Immune effects**
Concanavalin A	*Volvariellavolvacea*	Activating T lymphocyte
Ricin-B-like lectin (CNL)	*Clitocybenebularis*	Stimulating dendritic cells (DCs) and cytokines
TML-1, TML-2	*Tricholomamongolicum*	*Macrophages activator (TNF-α, Nitrite ions*

Cytokines and chemokines have a significant role in immunity and immunopathology in the human body during viral infection. They are the ﬁrst barrier of innate immunity that assists as a defense wall against the viral infection.

### Actinomycetes lectins

Actinohivin is a novel lectin isolated from *Longisporaalbida’s* cultural broth. The special sequence of this polypeptide consists of 114 amino acids and a 12.5 kDa molecular weight, which prevents the production of syncytium and the cytopathic effect of HIV.^53^ Similar biological activities were documented by Boyd et al for cyanovirin-N isolated from a cyanobacterium, such as cell-to-cell fusion abortion and HIV-1 infection.^39^ In contrast to cyanovirin-N, which can bind a single high-mannose glycan attached to the protein, Actinohivin is related to only glycoproteins with several high-mannose glycans. Also, Actinohivin has the potential to become a microbicide drug, as it can effectively prevent HIV-1 and HIV-2 with low IC_50_ values (2–110 nM).^54^

### Bacterial lectins

The development of lectins as microbicidal medicines is a new approach to the therapy for combating virus transmission. The recombinant expression of *Pseudomonas fluorescens* agglutinin in *E. coli* DE3 was biologically and structurally characterized. This agglutinin is a 14 kDa protein consisting of 133 residues in a tertiary structure homologous to the QAAH-lectin fold with 2 repeated sequences. This lectin demonstrated anti-HIV-1 activity in TZM-bl cells with 15 nM IC_50_.^55^ Similarly, *Myxococcus xanthus* hemagglutinin was recombinantly expressed in *E. coli* DE3 for biological and structurally characterized. The hemagglutinin is a 28 kDa protein consisting of 267 residues with 4 repeated sequences, and their crystal structures are disclosing QAAH-lectin fold. In TZM-bl cells with 12 nM IC50, this hemagglutinin showed anti-HIV-1 activity.^56^ However, *Burkholderiaoklahomensis* agglutinin has 276 residue proteins comprising an N-terminal 10 residue tail that does not exist in the other OAAH members. Like the OAAH members, *Burkholderiaoklahomensis*agglutinin showed anti-HIV-1 efficiency in TZM-bl cells with an IC_50_ range of 10–14 nM.^56^

### Archeal lectins

Lectins are known to engage in symbiosis, cell-cell interaction, antiviral antiproliferative, and mitogenic activities, and in the development of innate immunity.^57^ There are six eukaryotic and bacterial structural classes of lectins in archaea. However, there is a disconcertingly low number of archaea species where lectins can be identified. Twenty-one of archeal lectins having the b-propeller are 7-bladed, while sixteen have a B-trefoil, and seven have a legume lectin fold. The rest are assumed to be the lectin of type C, b prism I, and tachylectin folds.^58^ Till now, no data was recorded on the antiviral activity of archeal lectins.

### Antiviral lectin binding mechanisms

Viral recognition and entry depends on the affinity of the glycosylated envelope proteins to the host cells’ surface proteins.^59^ The spontaneous mutation and loss of oligosaccharide attachment sites prevent reading recognition of viral glycosylation of viral envelope glycoproteins.^60^ Continued challenges are there to enable broad-spectrum viral suppression support lectins as inhibitors of viral entry in the provision of prophylactic and potential viral infection therapeutics.^61^ The envelope proteins have a shared tertiary and quaternary architecture and function similarly. Antiviral lectins interact primarily with high-mannose glycan structures, which are added as post-translation modifications to the virus envelope proteins.^62^

The SARS-CoV spike protein S2 is responsible for allowing the host cell membrane to be combined with the virus. The HIV-1 gp41 and SARS-CoV S2 proteins have shown that there are similar structural motifs; (1) N-terminal leucine-isoleucine heptad recurring sequence on 913-1000 residues and (2) C-terminal leucine/isoleucine heptad recurrent motif on 1151-1185 residue. However, Wu Zhang and Yap showed that SARS-CoV S2 and HIV-1 gp41 share a very similar helix structure on 879–942 residues.^62^ It indicated that a similar membrane fusion process could be demonstrated by the two viruses. The N-linked oligosaccharide binding sites are found in gp41 and gp120 of HIV, and lectins interact with the enveloped protein (gp41 and gp120) complexes’ glycosylation moieties that prevent the conformational rearrangements necessary for HIV fusion.^62^

In general, lectins are classified based on their CRDs’ glycan identification, such as sugar specificity (mannose, glucose, N-acetyl galactosamine, etc). Additionally, some antiviral lectins have disulfide bonds, such as CV-N, scytovirin, and actinohivin that are within or between domains. Many lectins are solution dimers, trimers, or tetramers, and some are known to be monomers.^61^

The structural orientation of each CRD will add greater affinity for the oligosaccharides in lectins. In particular, some of them interact with the components of the mannoses; others interact with the entire glycan branches and some with the galactose core. The interactions in oligosaccharides between protein and/or lateral chain atoms and oxygen atoms are mainly derived from hydrogen bonding. Furthermore, they use the trio of aromatic residues that can supply the inner walls of the CRD. Besides, the side chains of tyrosine and tryptophan may sometimes interact directly with the oligosaccharides. Lectins of the *Oscillatoria agardhii* homologue family consist of residue side and back chains that participate in glycan hydrogen bonding and have an existing CRD sequence. The antiviral lectins counter viruses through a globally similar mechanism that binds viral glycoprotein envelopes into oligosaccharides and prevent viral fusion and entry through a steric barrier. This mechanism involves multiple CRDs in lectins for sufficient control, often relying on the cross-linkage of oligosaccharides on enveloped proteins by lectins.^61^

### Lectins or natural products as promising therapy against COVID -19

Recently, the cause of interaction with host C-type lectins was the O-glycan on the SARS-CoV-2 spike glycoprotein, which could exert an impact on the severity of infection and cause the uncontrolled immune response. Furthermore, the viral protein showed binding to glycol-conjugates expressed by colonized bacteria in lungs. These findings revealed implications for controlling and understanding the severity of COVID-19 infections. The capability of the carbohydrate-binding receptor of SARS-CoV-2 spike glycoprotein to binding to glycol-conjugates is important in the viral entry and modulation of the response of the immune system.^63^ This may explain the possibility of binding lectins in the plant, algae, bacteria and fungi to the viral glycoprotein*. Mannose-binding lectin can prevent viral interaction by blocking the viral binding to*dendritic cell-specific ICAM-grabbing non-integrin (mammalian expressed C- lectins). *Through the interference with the coronavirus entrance by binding to the high-mannose type N-glycans of SARS-CoV via the S protein, the viral attachment to target proteins and the host cell is prevented. The significance of lectins in viral defense is also demonstrated by the deficiency of MBL, which has been assumed as a susceptibility factor for SARS-CoV.*^64^

As shown before, griffithsin is a kind of lectin that binds to oligosaccharides on SARS-CoV spike glycoprotein.^12^ The spike proteins (S-protein) of covid-2019 and SARS-CoV share a very similar receptor-binding domain, which has a significant ACE2 binding affinity. A Molecular docking approach has been used to stimulate the supposed binding action between molecules. Previous research has stated the ability of several natural products, including neohesperidin, glycyrrhizin, and nobiletin, to block the binding between 2019-nCoV and its receptor, ACE2.^22,65^ Similarly, Naringin is now recorded in the protocol for diagnosis and treatment of the novel coronavirus.^66^ Besides, Das et al confirmed that rutin and hesperidin have anti-SARS-CoV-2 activity, under in vivo condition, by using molecular docking screening.^67^ Further molecular docking approach will be recommended for the investigation of the binding activity between natural lectins and SARS-CoV-2 and its receptor, ACE2.

### Interventions of lectins with other factors

The interventions of some plant lectins with sulfonamide were investigated by Butera et al, and the study showed that the β-lactosylamine amides and sulfonamides were practically active in inhibiting hemagglutination by lectins from the seeds of *Erythrina cristagalli* and were less active against agglutination by lectins from the seeds of *Ricinus communis*.^68^ Also, the in vivo and in vitro administration of glucocorticoids effect on lectins-induced proliferation of lymphocytes (concanavalin A, phytohaemagglutinin A) was examined in atopic dermatitis patients and in normal controls. In vitro dexamethasone administration has an important suppressive effect on lectin-induced blastogenesis in both normal controls and patients, as compared to impaired lymphocyte proliferation in vivo.^69^ Also, the prolonged anesthesia decreased the expression of mannose receptor, and caused alteration in the expression of receptors for several other sugars on murine peripheral blood polymorpho-nuclear leukocytes.^70^ Recent studies are needed to address the interventions of lectins with other factors.

### Lectins and other diseases

In general, Mannose Binding Lectins deficiency was shown to increase the susceptibility to recurrent infections and contributed to the pathogenesis of Behçet^’^s disease and Down^’^s syndrome. In Down syndrome patients, these deficiency increases the susceptibility to recurrent infections.^71^ Therefore, Nisihara et al recommended the future using of mannose-binding lectins therapy.^72^ Kolb et al also pointed to the suppressive effect of Concanavalin A on inbred mouse model type 1diabetes after low-dose streptozotocin.^73,74^

### Lectins as immune modulators

O’Keefe et alstudied the impact of griffithsin treated mice on immune response and the survival following subsequent re-exposure to the SARS virus. They found that the reason of mortality of SARS was due to the suppression of innate immune responses from the reduction of several cytokines in GRFT-treated mice, including IL-1, IL-6, G-CSF, MCP-1, and IL-12.^12^ Also, the robust cellular infiltrates after griffithsin treatment was due to increased immunogenicity of aggregation griffithsin/viral particles. The infiltrate modulates the protective immunity against SARS and recover the griffithsin treated animals, and the promotion of leukocyte infiltration has been recorded later the high concentration topical administration of griffithsin to rabbit cervical mucosa.^75^

## Conclusion

Till the time being, there are no selective active molecules on the market for the treatment of SARS-CoV-2. Lectins are carbohydrate-binding proteins, notably similar to individual molecules’ sugar groups. Lectins also mediate binding and attaching viruses, bacteria, and fungi to their intended targets. This review explains antiviral properties of lectins against COVID-19. For instance, all mannose-specific plant lectins have anti-coronaviruses properties except for garlic lectins. Also, griffithsin lectin, isolated from red algae, has noteworthy efﬁcacy against lethal human coronaviruses. Furthermore, lectins have several therapeutic applications, such as anticancer, antibacterial, antifungal activities, and also their suppressive effect on Type 1 diabetes. Lectins can also mediate drug targeting, which, because of its high specificity, has been positioned as one of the most pronounced drug targeting technologies. Fortunately, there are plenty of opportunities to improve antiviral drugs and SARS-CoV-2 vaccines, but rigorous efforts are needed to investigate such new antiviral therapy with more studies that explicitly target lectins. Lectins are the future hope of medicines but several research studies will be needed to improve its efficiency in promoting human health.

## Acknowledgments

Authors thankfully acknowledge Egyptian Drug Authority for positive support.

## Ethical Issues

Not applicable.

## Conflict of Interest

Authors declare no conflict of interest in this study.
